# Determination of 14 Circulating microRNAs in Swedes and Iraqis with and without Diabetes Mellitus Type 2

**DOI:** 10.1371/journal.pone.0086792

**Published:** 2014-01-30

**Authors:** Xiao Wang, Jan Sundquist, Bengt Zöller, Ashfaque A. Memon, Karolina Palmér, Kristina Sundquist, Louise Bennet

**Affiliations:** 1 Center for Primary Health Care Research, Lund University/Region Skåne, Sweden; 2 Stanford Prevention Research Center, Stanford University School of Medicine, Stanford, California, United States of America; Deutsches Krebsforschungszentrum, Germany

## Abstract

**Background:**

Recent reports suggest that immigrants from Middle Eastern countries are a high-risk group for type 2 diabetes (T2D) compared with Swedes, and that the pathogenesis of T2D may be ethnicity-specific. Deregulation of microRNA (miRNA) expression has been demonstrated to be associated with T2D but ethnic differences in miRNA have not been investigated. The aim of this study was to explore the ethnic specific expression (Swedish and Iraqi) of a panel of 14 previously identified miRNAs in patients without T2D (including those with prediabetes) and T2D.

**Methods:**

A total of 152 individuals were included in the study (84 Iraqis and 68 Swedes). Nineteen Iraqis and 14 Swedes were diagnosed with T2D. Expression of the 14 selected miRNAs (miR-15a, miR-20, miR-21, miR-24, miR-29b, miR-126, miR-144, miR-150, miR-197, miR-223, miR-191, miR-320a, miR-486-5p, and miR-28-3p) in plasma samples was measured by real-time PCR.

**Results:**

In the whole study population, the expression of miR-24 and miR-29b was significantly different between T2D patients and controls after adjustment for age, sex, waist circumference, family history of T2D, and a sedentary lifestyle. Interestingly, when stratifying the study population according to country of birth, we found that higher expression of miR-144 was significantly associated with T2D in Swedes (*OR* = 2.43, *p* = 0.035), but not in Iraqis (*OR* = 0.54, *p* = 0.169). The interaction test was significant (*p* = 0.017).

**Conclusion:**

This study suggests that the association between plasma miR-144 expression and T2D differs between Swedes and Iraqis.

## Background

Type 2 diabetes mellitus (T2D) is one of the most prevalent metabolic disorders worldwide. The prevalence is increasing dramatically and, in 2025, approximately 15% of people in the world are predicted to have T2D, impaired fasting glucose (IFG) or impaired glucose tolerance (IGT) [1]. The prevalence of T2D is higher among individuals living in the Middle East compared to those living in northern European countries [2]. In Sweden, the prevalence of T2D among immigrants from Middle Eastern countries is two to three times higher than that in native Swedes [2]. In addition, T2D patients from the Middle East have a younger age at onset, a higher prevalence of family history, and a more rapid decline in pancreatic β-cell function compared to Swedish patients, suggesting that they may have a different form of T2D compared with Swedish patients, and that the pathogenesis of T2D may be ethnicity specific [3]. Several genes were recently detected as relevant in T2D by genome-wide association studies (GWASs) [4], [5]. However, their usefulness as biomarkers and predictors of disease remains uncertain [6], and additional studies to identify other potential biomarkers and predictors are needed.

MicroRNAs (miRNAs) are a class of endogenous, small (21–23-nucleotide), non-coding single-stranded RNAs that negatively regulate gene expression by binding to the 3′-untranslated regions (3′-UTRs) of target mRNA molecules [7]. MiRNAs have been shown to play essential roles in various biological processes, including cell development and proliferation, apoptosis, metabolism, and cell differentiation [8]. The majority of miRNAs are expressed intracellularly. However, numerous miRNAs have recently been found in the extracellular space, including the blood and other body fluids [9]. Circulating miRNAs are associated with proteins, microvesicles and/or lipoprotein complexes [10]–[12]. In vitro studies indicate that circulating miRNAs transported by microvesicles or lipoprotein complexes can be transferred in an active form to recipient cells, and are involved in cell-to-cell communication [12]–[14]. Circulating miRNAs are relatively stable, being resistant to nuclease digestion, and can be measured reproducibly, which make them attractive as potential biomarkers.

The idea of using miRNAs in blood as biomarkers is quite new and was first proposed for improving diagnoses of cancer [15], [16]. Studies have shown that by mediating gene expression, miRNAs strongly influence glucose balance and the development of diabetes [17]. Recent studies have analyzed the profiles of miRNAs in blood to evaluate their roles in the development and progression of T2D [18]–[21]. However, the results were inconsistent, possibly as a consequence of study populations being ethnically and genetically heterogeneous, or because of differences in diagnostic criteria and treatment and/or protocols for sample preparation and miRNA analysis. For instance, the higher prevalence of family history of T2D among subjects born in Iraq compared to native Swedes supports a stronger genetic component in this ethnic group compared to Swedes [22]. The prevalence of family history in immigrants from the Middle East diagnosed with diabetes is also higher than in Swedes diagnosed with diabetes [3], [22] [23]. This could support a stronger genetic burden amongst diabetes patients born in Iraq, but information on type 2 diabetes susceptibility genes in populations from the Middle East are scarce [3]. Further, it is still not known whether the expression of different miRNAs varies according to ethnic background. Increased knowledge in this novel research area would further contribute to our understanding of the complex mechanisms that influence the development of T2D in groups with different ethnic origins.

The aim of this survey was to study differences in plasma miRNA expression between patients with and without T2D and in native Swedes and Iraqis by analyzing a panel of 14 previously identified miRNAs [19], [21]. The choice of these 14 miRNAs is based on previous studies showing that these miRNAs are associated with T2D-related outcomes. These 14 selected miRNAs have been reported to be involved in insulin biosynthesis, insulin signaling, insulin resistance, regulation of glucose-induced gene expression in diabetes, and lipid metabolism [19], [21], [24]–[27], all of which are associated with the pathogenesis of T2D.

## Materials and Methods

### Ethical statement

The study was performed according to the declaration of Helsinki [28]. The ethical committee at Lund University approved the study (approval no. 2009/36) and written informed consent was given by all the participants in the study.

### Study population

The present study was performed as part of the larger MEDIM (impact of Migration and Ethnicity on Diabetes In Malmö) study, a population-based study of the prevalence of T2D in an urban Swedish population of Swedish and Iraqi origin [22]. Female and male adult subjects (45 to 65 years of age) born in Sweden or Iraq were randomly selected from the census register for the city of Malmö and were recruited to the study between February 1 and March 2010. All participants were selected from a single deprived neighborhood and were diagnosed according to the same criteria, guaranteeing comparability between the two ethnic groups. After sample quality (exclusion of hemolysis [29], [30]) and quantity (plasma<250 µl) control, 152 individuals were included (84 Iraqis and 68 Swedes). Each participant signed a written informed consent form, filled in a questionnaire, and provided blood samples [22]. All participants except for those previously diagnosed with diabetes underwent an oral glucose tolerance test (OGTT), performed as previously described [22]. Normal glucose tolerance was defined as a fasting glucose level of <6.1 mmol/L and a plasma glucose level of <7.8 mmol/L 2 h after a 75 g OGTT. IFG was defined as a fasting plasma glucose level of ≥6.1 mmol/L and <7.0 mmol/L and a 2-h plasma glucose level of <7.8 mmol/L. IGT was defined as a fasting plasma glucose level of <7.0 mmol/L and a 2-h plasma glucose level of ≥7.8 mmol/L and <11.1 mmol/L. T2D was confirmed by a fasting plasma glucose level of ≥7.0 mmol/L and/or a 2-h plasma glucose level in the OGTT of ≥11.1 mmol/L [22].

### Plasma collection

Blood samples were collected after a 10-h overnight fast. Four mLs of whole blood was collected from each participant in EDTA tubes. Blood samples were centrifuged at 2,000 g for 10 min at 4°C, and the plasma was then aliquoted and stored at −80°C before further processing. Blood samples were processed and plasma frozen within 4 h of collection.

### RNA extraction

Arroyo et al found that there are two fractions of circulating miRNAs, protein-rich and exosome-rich fractions. MiRNAs isolated from plasma samples in our study account for 90% of the total circulating miRNAs [11]. Total RNA was isolated from plasma samples using Qiagen miRNeasy Mini Kit (Qiagen GmbH, Germany) according to the manufacture's protocol, with minor modifications. Briefly, QIAzol master mix (800 µL of QIAzol solution plus 1.25 µL of 0.8 µg/µL MS2 RNA) was added to 200 µL of human plasma, vortex-mixed, and incubated at room temperature for 5 min. The use of a carrier ensures the highest and most consistent isolation from serum and plasma samples. After phase separation by addition of chloroform and centrifugation, the aqueous phase was mixed with ethanol and then loaded into a mini spin column. The column was then washed with wash solution. The final elution volume was 50 µL. RNA concentrations were determined using a NanoDrop ND-1000 spectrophotometer (Thermo Scientific, Wilmington, USA), ranging from 19 to 23 ng/µL. The samples were either stored at −80°C or further processed.

### Reverse transcription

Reverse transcription (RT) was carried out using a Universal cDNA synthesis kit (Exiqon, Denmark) according to the manufacturer's instructions. Plasma contains very low amounts of RNA. We used a carrier RNA (MS2 RNA) during extraction to ensure the highest and most consistent isolation from serum and plasma samples. Our measurements would then only reflect the carrier RNA. Therefore, equal volumes of RNA preparation, rather than equal RNA amounts, was used as input in the cDNA synthesis [19], [31]. Each reaction consisted of 4 µL of RNA and 16 µL of RT master mix (4 µL of 5× reaction buffer, 9 µL of nuclease-free water, 2 µL of enzyme mix, and 1 µL of synthetic spike in (a control for the quality of the cDNA synthesis reaction and the PCR). Reaction mixtures (20 µL) were incubated for 60 min at 42°C and for 5 min at 95°C, and were then held at 4°C in a MyCycler thermal cycler (Bio-Rad Laboratories, CA, USA). The resulting reverse transcription reaction product was stored at −20°C before analysis.

### Quantitative real-time PCR

Quantitative real-time PCR (qPCR) was carried out in 384-well plates using the CFX384™ real-time PCR detection system (Bio-Rad, Laboratories). qPCR was performed in duplicate for each miRNA, and a non-template control was included for each primer set. To minimize inter-assay variation, all samples were analyzed for a particular miRNA on the same plate. The 10 µL PCR reaction mixture included 5 µL of SYBR green master mix (Universal RT, Exiqon), 1 µL of MicroRNA LNATM PCR primer mix (Exiqon), and 4 µL of cDNA template (diluted 1∶80). Amplification was achieved by incubation for 10 min at 95°C, followed by 40 cycles of 95°C for 10 s and 60°C for 1 min. The threshold cycle (Ct) values were obtained from amplification of all miRNAs. Ct values >36 were considered to indicate either miRNA levels below the technique detection limit or that they were absent. We assessed the RNA quality in all our samples before we ran selected miRNAs, i.e., miR-93, miR-103, miR-191, miR-423-3p, miR-425 and miR-451. These miRNAs are commonly found and stably expressed in plasma [32] and they were quite stable across all samples with a coefficient of variation varying between of 2.5% and 4.5% ( Table S1). MiR-451 is highly expressed in red blood cells and was therefore chosen as a candidate for evaluating hemolysis in the present study [29]. To adjust for differences in the amount of total RNA between samples, Ct values were normalized to one reference miRNA. In addition to the above-mentioned miRNAs, we also measured miR-454and RNAU6b expression [19], [33] in all samples. MiR-454 and RNAU6b were not detected in any samples. All the other miRNAs were included for further investigation using the currently available major computational programs (geNorm, Normfinder, BestKeeper, and the comparative ΔCt method) to compare and rank these candidate miRNAs [33]. MiR-425 was stable and abundant in the plasma, and its expression varied modestly between samples, with a coefficient of variation of 2.5%. Therefore, miR-425 was used as an internal control to adjust for differences in the amount of total RNA between samples. MiR-425 was also used as an internal control in a recently published article [34]. Ct values were normalized to miR-425 and transformed into quantities (relative expression) using the following equation: relative expression = 2^−ΔCt^, where Ct is the threshold cycle for a sample and ΔCt = Ct_miR of interest_−Ct_miR-425_. To compare the miRNA level, the expression of individual miRNA in diabetic samples is compared to expression of the same miRNA in non-diabetic samples. The results are shown as fold change (FC). Fold change is a measure describing how much a quantity changes going from an initial to a final value. FC of individual miRNAs in diabetic cases relative to non-diabetic cases were calculated as the mean log-transformed miRNA expression in the T2D group divided by the mean expression in the control (non-diabetes) group. We used 1.5 as the FC cut-off, which is based on several previous profiling studies confirming that subtle changes in miRNA expression in a 1.5-fold difference can have a significant impact on the biology of the cell [35]. In addition, a power analysis revealed that a fold change of 1.46 could be detected at a significance level of 5% and with a power of 80% in the studied population.

### Statistical analysis

Ethnic differences in sample characteristics were tested using chi-square tests for dichotomous variables, T-tests for continuous variables' and Wilcoxon's rank-sum test for all miRNAs (due to the skewed distributions). Logistic regression was used to examine the associations between miRNA levels and hyperglycemia (T2D versus the control group). All miRNAs were transformed with a natural logarithm function to obtain an approximate normal distribution and were scaled to have the same mean and standard deviation. First, univariate analysis was performed for each miRNA and thereafter the associations were adjusted for male sex (yes/no), age (continuous), waist circumference (continuous), family history of T2D (yes/no), and sedentary lifestyle (yes/no), which all have been shown to be associated with T2D [22]. All analyses were then stratified by ethnicity to examine potential differences between individuals born in Sweden and those born in Iraq. The associations were also examined by including an interaction term between miRNA and country of birth in the model. Finally, Pearson's correlations between logarithmically transformed miRNAs and insulin resistance parameters, i.e., insulin sensitivity index (ISI) and homeostatic model assessment-insulin resistance (HOMA-IR), were conducted. STATA version 12 (StataCorp LP) was used for all statistical analyses.

## Results

The clinical characteristics and miRNA distributions of the total study population are presented in Table 1. The Swedish-born participants were on average older compared to ethnic Iraqis. The prevalence of family history of T2D was significantly higher in Iraqi participants than in Swedish participants. The Swedish participants were significantly taller than the Iraqi participants. There were no significant differences in sex distribution, waist circumference, weight, sedentary lifestyle, or median expression of all the 13 selected miRNAs between the two groups. MiR-20b was not detected (Ct values were >36) in more than 20% of patients. Due to the expression patterns of miRNAs in individuals with IFT/IGT being similar to those in individuals with normal glucose tolerance (data not shown), we combined them as a control group in subsequent analyses.

**Table 1:** Characteristics of the study population stratified by country of birth.

	All			Iraq			Sweden			
Variables	N	Mean/%	SD	N	Mean/%	SD	N	Mean/%	SD	p-value^a^
Age	152	55.76	5.80	84	54.64	6.19	68	57.15	4.99	0.008
Waist circumference	151	98.95	12.11	83	99.45	12.25	68	98.34	11.99	0.578
Weight (kg)	140	79	14	80	79	14	60	79	14	0.90
Height (cm)	140	167	11	80	163	9	60	173	10	0.000
Sex										
Male	83	55		45	54		38	56		0.776
Female	69	45		39	46		30	44		
T2D										
Yes	33	22		19	23		14	21		0.763
No	119	78		65	77		54	79		
Family history										
Yes	55	36		41	49		14	21		0.000
No	74	49		26	31		48	71		
Sedentary lifestyle										
Yes	96	63		54	64		42	62		0.288
No	47	31		22	26		25	37		
MiRNAs										
mir-15a	152	12.77	7.61	84	12.64	9.36	68	13.04	7.11	0.542
mir-21	152	19.23	9.15	84	19.03	8.63	68	19.91	8.33	0.127
mir-24	152	4.10	1.88	84	4.00	1.87	68	4.17	1.87	0.368
mir-29b	152	0.18	0.08	84	0.18	0.09	68	0.17	0.08	0.461
mir-126	152	8.97	4.34	84	8.94	4.25	68	9.16	4.27	0.633
mir-144	152	3.43	3.28	84	3.49	3.73	68	3.28	2.93	0.525
mir-150	152	2.05	1.80	84	2.05	2.05	68	2.06	1.61	0.966
mir-197	152	0.41	0.22	84	0.43	0.28	68	0.40	0.15	0.746
mir-223	152	19.77	15.03	84	20.83	14.98	68	19.29	14.52	0.198
mir-191	152	0.37	0.21	84	0.38	0.20	68	0.35	0.23	0.990
mir-320a	152	8.11	3.69	84	8.11	3.92	68	8.17	3.71	0.418
mir-486-5p	152	17.15	18.85	84	17.76	20.20	68	16.97	15.60	1.000
mir-28-3p	152	0.22	0.11	84	0.22	0.11	68	0.23	0.11	0.945

Age,waist circumference, weight and height were presented with mean and SD and tested with T-test.

Sex, hyperglycemia, family history and sedentary lifestyle were presented with % and tested with Chi2-test.

All miRNAs were presented with median and IQR and tested with Wilcoxon rank-sum test.

a P-value for test between Iraq and Sweden.

In Table 2 we present the miRNA distributions by country of birth and T2D diagnosis. In a total of 9 miRNAs the expression differed between patients with T2D as compared with those without T2D. When the study population was stratified by country of birth, miR-144, miR-150 and miR-486-5p showed different patterns between Iraqi and Swedish participants; miR-144 and miR-486-5p showed significant higher expressions in T2D compared to the control group in Swedish participants only, whereas miR-150 showed significant higher expressions in T2D compared to the control group in Iraqis only.

**Table 2:** MiRNA by T2D and origin.

	All			Iraq			Sweden		
Variables	Control	T2D	p-value^a^	Control	T2D	p-value	Control	T2D	p-value
	(n = 119)	(n = 33)		(n = 65)	(n = 19)		(n = 54)	(n = 14)	
mir-15a	12.13	17.15	0.001	12.13	15.45	0.049	12.30	19.43	0.007
mir-21	18.90	24.08	0.001	17.75	24.08	0.002	19.16	23.81	0.086
mir-24	3.92	4.72	0.001	3.92	4.53	0.011	3.90	4.81	0.033
mir-29b	0.16	0.21	0.001	0.17	0.21	0.071	0.16	0.22	0.004
mir-126	8.63	11.08	0.005	8.51	10.48	0.019	8.75	11.28	0.143
mir-144	3.27	4.96	0.051	3.46	4.53	0.657	3.11	5.35	0.020
mir-150	1.89	2.75	0.000	1.88	4.06	0.001	1.96	2.40	0.110
mir-197	0.41	0.39	0.837	0.42	0.49	0.140	0.41	0.37	0.150
mir-223	20.11	17.75	0.739	21.86	17.63	0.638	19.29	18.86	0.891
mir-191	0.36	0.41	0.137	0.37	0.41	0.300	0.35	0.42	0.328
mir-320a	7.52	10.27	0.000	7.46	9.13	0.006	7.78	10.41	0.027
mir-486-5p	16.80	30.48	0.006	16.34	21.71	0.103	16.85	35.54	0.026
mir-28-3p	0.22	0.24	0.349	0.22	0.22	0.642	0.23	0.26	0.355

All miRNAs were presented with median and tested with Wilcoxon rank-sum test.

Control = Normal+IFG/IGT.

a P-value for test between control and T2D.

Table 3 shows unadjusted odds ratios (ORs) for a 1-SD unit increase of each miRNA. Of the 13 miRNAs we examined, 9 were associated with increased odds of diabetes in the total population. However, only miR-24 (*OR* = 2.39, *p* = 0.008) and miR-29b (*OR* = 1.93,*p* = 0.020) remained significant after adjustment for age, sex, waist circumference, family history of T2D, and sedentary lifestyle (Table 4), but they had low FC with 1.18 and 0.86, separately. In addition, we tested for all associations by adjusting for body mass index (BMI) instead of waist circumference, and the results for BMI were almost identical (data not shown in tables).

**Table 3:** Standardized unadjusted logistic regression of T2D versus control, stratified by country of birth.

	All			Iraq			Sweden		
Variables	OR^a^	p-value	95% CI	OR	p-value	95% CI	OR	p-value	95% CI
mir-15a	2.36	0.000	1.52–3.65	1.89	0.022	1.10–3.26	3.40	0.003	1.50–7.70
mir-21	2.09	0.001	1.37–3.19	2.82	0.002	1.46–5.47	1.72	0.050	1.00–2.96
mir-24	2.06	0.002	1.31–3.25	2.14	0.012	1.18–3.90	1.99	0.058	0.98–4.06
mir-29b	1.94	0.003	1.25–3.02	1.47	0.156	0.86–2.51	3.21	0.007	1.39–7.44
mir-126	1.87	0.003	1.23–2.83	1.84	0.024	1.08–3.14	1.92	0.055	0.99–3.72
mir-144	1.49	0.042	1.01–2.18	1.03	0.895	0.63–1.68	2.86	0.005	1.36–6.01
mir-150	2.11	0.000	1.39–3.18	2.22	0.003	1.32–3.74	1.90	0.066	0.96–3.79
mir-197	1.06	0.770	0.72–1.57	1.45	0.165	0.86–2.45	0.56	0.114	0.27–1.15
mir-223	0.99	0.941	0.67–1.45	0.92	0.767	0.54–1.57	1.05	0.875	0.59–1.87
mir-191	1.39	0.104	0.93–2.07	1.29	0.365	0.74–2.24	1.52	0.164	0.84–2.73
mir-320a	2.32	0.000	1.53–3.53	2.29	0.003	1.32–3.95	2.41	0.008	1.26–4.63
mir-486-5p	2.05	0.001	1.37–3.08	1.63	0.068	0.96–2.76	2.80	0.003	1.41–5.57
mir-28-3p	1.30	0.207	0.87–1.93	1.26	0.423	0.71–2.24	1.33	0.326	0.75–2.34

Control = Normal+IFG/IGT.

All miRNAs were log-transformed.

a Odds ratios for a 1-SD unit increase of miRNA.

**Table 4:** Standardized adjusted logistic regression of T2D versus control, stratified by country of birth.

	All			Iraq			Sweden		
Variables	OR^a^	p-value	95% CI	OR	p-value	95% CI	OR	p-value	95% CI
mir-15a	1.60	0.096	0.92–2.77	0.99	0.977	0.39–2.52	2.39	0.049	1.00–5.70
mir-21	1.61	0.090	0.93–2.80	2.26	0.087	0.89–5.76	1.41	0.340	0.69–2.87
mir-24	2.39	0.008	1.26–4.54	4.22	0.009	1.44–12.36	1.57	0.325	0.64–3.85
mir-29b	1.93	0.020	1.11–3.36	1.63	0.216	0.75–3.54	2.04	0.117	0.84–5.00
mir-126	1.40	0.244	0.79–2.48	1.57	0.225	0.76–3.23	1.05	0.916	0.40–2.79
mir-144	1.20	0.485	0.72–2.01	0.54	0.169	0.22–1.30	2.43	0.035	1.07–5.55
mir-150	1.41	0.217	0.82–2.45	1.47	0.282	0.73–2.99	1.07	0.895	0.42–2.72
mir-197	1.11	0.699	0.66–1.86	1.87	0.106	0.87–4.01	0.38	0.072	0.13–1.09
mir-223	1.19	0.569	0.66–2.14	1.03	0.951	0.44–2.37	1.17	0.742	0.46–2.97
mir-191	0.99	0.969	0.57–1.72	0.94	0.886	0.41–2.16	1.01	0.975	0.45–2.27
mir-320a	1.53	0.139	0.87–2.70	1.60	0.267	0.70–3.66	1.56	0.274	0.70–3.45
mir-486-5p	1.29	0.362	0.75–2.23	0.67	0.419	0.26–1.76	2.00	0.079	0.92–4.35
mir-28-3p	1.19	0.512	0.70–2.02	1.17	0.667	0.57–2.44	1.17	0.726	0.49–2.75

Control = Normal+IFG/IGT.

Adjusted for sex, age, waist circumference, family history of T2D and sedentary lifestyle.

All miRNAs were log-transformed.

a Odds ratios for a 1-SD unit increase of miRNA.

Similar analyses were conducted for Iraqi and Swedish participants separately. For Iraqis, only miR-24 (Table 4) was significantly associated with T2D (*OR* = 4.22, *p* = 0.009) but with a low FC (1.2), after adjustment for the above confounding factors. In native Swedes, the miR-15a and miR-144 independently of other risk factors increased the odds of T2D (miR-15a *OR* = 2.39, *p* = 0.049 and miR-144 *OR* = 2.43, *p* = 0.035) (Table 4), with FCs of 1.26 and 1.58, respectively. With a FC cut-off of 1.5, only miR-144 was significantly up-regulated in Swedish participant with T2D as compared to those without T2D. To further examine the potential differences in the associations between miRNAs and T2D between the two groups, we performed an interaction test.

We found a significant interaction between miR-144 and country of birth (*OR* = 0.24, *p* = 0.017) (Table 5). The odds ratio for a 1-SD unit increase of miRNA was 0.24 times lower in Iraqis than in Swedes.

**Table 5:** Interactions between miRNA and country of birth.

Variables	OR	p-value	95% CI
mir-15a*Iraq	0.46^a^	0.190	0.14–1.47
mir-21*Iraq	1.38	0.543	0.49–3.95
mir-24*Iraq	2.69	0.145	0.71–10.17
mir-29b*Iraq	0.89	0.833	0.29–2.71
mir-126*Iraq	1.27	0.659	0.44–3.61
mir-144*Iraq	0.24	0.017	0.08–0.78
mir-150*Iraq	1.36	0.554	0.49–3.79
mir-197*Iraq	3.88	0.022	1.21–12.42
mir-223*Iraq	0.92	0.886	0.31–2.77
mir-191*Iraq	0.80	0.692	0.27–2.37
mir-320a*Iraq	0.95	0.925	0.33–2.77
mir-486-5p*Iraq	0.39	0.095	0.13–1.18
mir-28-3p*Iraq	0.94	0.908	0.33–2.66

Adjusted for sex, age, waist circumference, family history of T2D and sedentary lifestyle.

All miRNAs were log-transformed.

a The odds ratio for a 1-SD unit increase of miRNA is 0.46 times lower in Iraqis than in Swedes.

We also performed correlation tests between the 14 miRNAs and parameters of insulin resistance, as estimated by ISI and HOMA-IR (see Table 6 and 7). The overall pattern showed, as in the logistic regression analyses, that the correlations seemed to be stronger in Swedes than in Iraqis.

**Table 6:** Correlations between miRNA and Insulin Sensitivity Index (ISI), stratified by country of birth.

	All		Iraq		Sweden	
Variables	ρ^a^	p-value^b^	ρ	p-value	ρ	p-value
mir-15a	−0.11	0.21	0.04	0.74	−0.27	0.04
mir-21	−0.11	0.23	−0.05	0.68	−0.20	0.13
mir-24	−0.20	0.03	−0.07	0.58	−0.39	0.003
mir-29b	−0.19	0.03	0.05	0.69	−0.40	0.002
mir-126	−0.25	0.005	−0.21	0.10	−0.34	0.009
mir-144	−0.07	0.46	0.11	0.40	−0.26	0.05
mir-150	−0.19	0.04	−0.14	0.26	−0.25	0.06
mir-197	0.12	0.19	0.27	0.03	−0.11	0.41
mir-223	−0.12	0.20	0.09	0.49	−0.29	0.03
mir-191	−0.15	0.11	0.03	0.83	−0.31	0.02
mir-320a	−0.09	0.35	0.07	0.60	−0.25	0.06
mir-486-5p	−0.10	0.26	0.07	0.60	−0.27	0.04
mir-28-3p	−0.05	0.61	0.04	0.75	−0.13	0.32

All miRNAs were log-transformed.

ISI was log-transformed.

a Pearson's correlation coefficient.

b P-value for test of correlation coefficient different from zero.

**Table 7:** Correlations between miRNA and Homeostasis Model of Assessment - Insulin Resistance (HOMA-IR), stratified by country of birth.

	All		Iraq		Sweden	
Variables	ρ^a^	p-value^b^	ρ	p-value	ρ	p-value
mir-15a	0.12	0.15	0.05	0.67	0.22	0.07
mir-21	0.17	0.04	0.19	0.08	0.19	0.12
mir-24	0.23	0.004	0.16	0.15	0.37	0.002
mir-29b	0.23	0.004	0.08	0.46	0.42	0.0003
mir-126	0.27	0.0008	0.26	0.02	0.30	0.01
mir-144	0.01	0.87	−0.09	0.40	0.16	0.19
mir-150	0.27	0.001	0.28	0.01	0.24	0.05
mir-197	−0.003	0.97	−0.09	0.44	0.16	0.19
mir-223	0.08	0.31	−0.02	0.82	0.19	0.11
mir-191	0.12	0.13	0.02	0.85	0.24	0.04
mir-320a	0.15	0.06	0.10	0.37	0.25	0.04
mir-486-5p	0.10	0.23	0.03	0.76	0.19	0.13
mir-28-3p	0.12	0.14	0.06	0.59	0.19	0.11

All miRNAs were log-transformed.

HOMA-IR was log-transformed.

a Pearson's correlation coefficient.

b P-value for test of correlation coefficient different from zero.

## Discussion

To our knowledge, this is the first study to investigate the expression of a panel of miRNAs in groups of subjects with different ethnic origins as well as with and without T2D. The key finding of the present study is that plasma expression of miR-144 seems to be ethnicity- related, with higher expression in only native Swedes diagnosed with T2D.

In recent years, the notion of circulating miRNA as biomarkers for various diseases has been thoroughly explored. Studies have reported that circulating miRNAs are released from specific cells and transferred to recipient cells to exert their function [36]. Furthermore, aberrant expression of circulating miRNA was demonstrated to correspond to tissue injury [37], which raises the possibility of blood-based miRNA profiles as fingerprints of diseases. Recent studies analyzed miRNA profiles in serum, plasma, and blood cells in order to develop new approaches for predicting the development and progression of diabetes mellitus [18]–[20]. Although many studies have examined the expression patterns of various miRNAs in T2D, the results were quite inconsistent, suggesting that a large cohort of patients is needed in further studies to guarantee sufficient power. In addition, study protocols, including those for sample preparation, RNA extraction, miRNA analysis, and statistical analyses, should be standardized to allow the results from different studies to be compared.

Evidence shows that the clinical characteristics of T2D patients from the Middle East are different than those of patients from Sweden [22], suggesting ethnicity-specific pathogenesis. The expression of miRNA has been found to vary according to patients race/ethnicity [38] in other diseases, such as colorectal cancer [38]. However, whether the expression of miRNAs is ethnicity related in diabetes-related outcomes is still unknown. Our study indicates that the association between circulating miR-144 expression and T2D may be ethnicity dependent, with only Swedish patients showing positive associations. Increased miR-144 expression was not associated with T2D in ethnic Iraqis. The level of miR-144 has been found to be increased in the plasma of T2D patients and miR-144 has also been reported to be involved in the regulation of insulin sensitivity in muscle tissue [39]. Increased expression of miR-144 has been found to directly downregulate insulin receptor substrate 1 (*IRS1*), which is involved in insulin signaling at both the mRNA and protein level [21], [39]. One possible explanation for the difference in association between Iraqis and Swedes may be related to differences in lifestyle, including dietary differences. It is known that miRNAs from the food can regulate the expression of target genes in mammals [40]. In addition, a previous study suggested that the higher prevalence of obesity in Iraqis than in the Swedish population [41] and the lower physical activity levels in Iraqis [22], [42]may also influence the expression of miRNA [43]. However, no differences were noted in waist circumference and sedentary lifestyle between the two population groups in our study, suggesting that these variables play a minor role in the observed differences of expression of miRNA in this study. Further investigations are required to confirm and explore the underlying mechanism of potential ethnicity-specific associations between miR-144 and T2D in larger-scale studies (both in the number of samples and in the number of miRNAs). In addition, it will be of interest to examine whether the expression of exosome miRNA, and not only plasma miRNA, is ethnically related in patients with T2D.

Two other miRNAs, i.e., miR-150 and miR-486-5p, were also found to have different expression patterns in Iraqi and Swedish participants. MiR-150 has previously been reported to be up-regulated in insulin sensitivity tissues and to target the glucose transporter-4 (GLUT4) in the final stage of insulin signaling [21]. MiR-486-5p regulates the expression of silent information regulator 1 (SIRT1) [44], and SIRT1 expression is correlated with insulin sensitivity and energy expenditure in type 2 diabetic patients [45]. However, associations between miR-150 and miR-486-5p and T2D were not significant after adjustment for age, sex, waist circumference, family history of T2D, and sedentary lifestyle.

The other 12 miRNAs that have previously been associated with T2D were found to be associated with diabetes only in the univariate analysis in the present study and not in the multivariate analysis [19]. One possible reason could be the low sample size. Alternatively, the difference in ethnicity could also explain these differences. RNA isolation procedures are not consistent between studies, which means that they are not directly comparable. In addition, there is still a lack of a consensus for a reference miRNAs for qPCR analysis of plasma miRNAs that is used to normalize plasma miRNA levels [19]. However, this is unlikely to have an impact on our analysis of potential ethnic differences because the same isolation procedure was used for both ethnic groups.

In summary, we examined the expression of 14 miRNAs that has previously been shown to be associated with T2D-related outcomes, and found that the association between miR-144 expression and T2D was different between Swedes and Iraqis. This study is a proof of principle for a possible association between plasma miRNA expression and ethnical background for T2D. Further studies are needed to validate these findings and examine which pathways may lay behind the observed ethnic differences in the present study.

## Supporting Information

Table S1 **Mean of quantification cycle and coefficient of variation of selected miRNAs.** MiR-93, miR-103, miR-191, miR-423-3p, miR-425 and miR-451 are stably expressed across all samples with a coefficient of variation varying between of 2.5% and 4.5%.
(DOC)
