# Supplementary material for: Determination of 14 Circulating microRNAs in Swedes and Iraqis with and without Diabetes Mellitus Type 2
Source: PLoS One. 2014 Jan 30;9(1):e86792. doi: 10.1371/journal.pone.0086792 (PMC3907562; doi:10.1371/journal.pone.0086792)
Supplement: Table S1 — Mean of quantification cycle and coefficient of variation of selected miRNAs. MiR-93, miR-103, miR-191, miR-423-3p, miR-425 and miR-451 are stably expressed across all samples with a coefficient of variation varying between of 2.5% and 4.5%. (DOC) [file pone.0086792.s001.doc]

**Table S1. Mean of quantification cycle and coefficient of variation of selected miRNAs.**

| miRNAs | miR-93 | miR-103 | miR-191 | miR-423 | miR-425 | miR-451 |
| --- | --- | --- | --- | --- | --- | --- |
| Mean of quantification cycle (Cq) | 29.9 | 31.7 | 32.9 | 31.7 | 31.8 | 25.3 |
| Coefficient of variation (CV %) | 3 | 2.6 | 3.0 | 2.9 | 2.5 | 4.5 |
